# Phase II trial of S-1 plus leucovorin in patients with advanced gastric cancer and clinical prediction by S-1 pharmacogenetic pathway

**DOI:** 10.1007/s00280-016-3209-1

**Published:** 2016-12-02

**Authors:** Ming-ming He, Dong-sheng Zhang, Feng Wang, Zi-xian Wang, Shu-qiang Yuan, Zhi-qiang Wang, Hui-yan Luo, Chao Ren, Miao-zhen Qiu, Ying Jin, De-shen Wang, Dong-liang Chen, Zhao-lei Zeng, Yu-hong Li, Yang-yang He, Yuan-tao Hao, Pi Guo, Feng-hua Wang, Yi-xin Zeng, Rui-hua Xu

**Affiliations:** 1 State Key Laboratory of Oncology in South China, Collaborative Innovation Center for Cancer Medicine, Sun Yat-sen University Cancer Center, 651 Dong Feng Road East, Guangzhou, 510060 China; 2 Beijing Key Laboratory of Drug Targets Identification and Drug Screening, Institute of Materia Medica, Chinese Academy of Medical Sciences, Peking Union Medical College, Beijing, China; 3 Department of Medical Statistics and Epidemiology, School of Public Health, Sun Yat-sen University, Guangzhou, China; 4 Beijing Hospital, Beijing, China

**Keywords:** Advanced gastric cancer, S-1, Leucovorin, Pharmacogenetic

## Abstract

**Background:**

The first one-arm phase II trial aimed to evaluate and predict efficacy and safety of S-1 plus oral leucovorin (S-1/LV) as first-line chemotherapy for patients with advanced gastric cancer (AGC), using S-1 pharmacogenetic pathway approach.

**Patients and methods:**

A total of 39 patients orally took S-1 at conventional dose and LV simultaneously at a dose of 25 mg twice daily for a week, within a 2-week cycle. The primary endpoint was overall response rate (ORR), while the secondary endpoints were progression-free survival (PFS), time to failure (TTF), overall survival (OS), disease control rate (DCR), and adverse events (AEs). Peripheral blood was sampled prospectively for baseline expression of dihydropyrimidine dehydrogenase (DPD), orotate phosphoribosyltransferase (OPRT), thymidine phosphorylase (TP), and thymidylate synthase (TS), CYP2A6 gene polymorphisms, and 5-FU pharmacokinetics.

**Results:**

The ORR and DCR were 41.0 and 76.9%. The median PFS, TTF, and OS were 4.13, 3.70, and 11.40 months. Grade 3–4 AEs occurred in only 13 patients, and grade 4 AEs occurred in only 1 of them. High OPRT/TS and peritoneal metastasis (vs. liver metastasis) independently predicted responding. High OPRT/DPD independently predicted grade 3–4 AEs. High AUC_0–24h_ of 5-FU and metastatic/recurrent sites ≤2 (vs. >3) independently predicted prolonged PFS. Low baseline plasmic DPD independently predicted prolonged OS.

**Conclusions:**

Two-week, oral S-1/LV regimen demonstrated promising efficacy and safety as first-line chemotherapy for AGC.

**ClinicalTrials.gov identifier:**

NCT02090153

**Electronic supplementary material:**

The online version of this article (doi:10.1007/s00280-016-3209-1) contains supplementary material, which is available to authorized users.

## Background

A globally accepted standard regimen for advanced gastric cancer (AGC) has not been established. S-1, a fourth-generation oral fluorouracil (5-FU), has opened new perspectives with simplicity and convenience over the traditional backbone of chemotherapy for AGC, 5-FU.

S-1 contains tegafur/gimeracil/oteracil. Gimeracil reduces the degradation of 5-FU by inhibiting dihydropyrimidine dehydrogenase (DPD), and oteracil improves its gastrointestinal tolerability by inhibiting orotate phosphoribosyltransferase (OPRT). S-1 plus cisplatin is recommended as standard treatment by the Japanese Gastric Cancer Association and has been approved in Asian and European countries [1]. However, many patients cannot tolerant the toxicity of the widely accepted S-1 plus platinums [2].

One of the potential partners of S-1 is leucovorin (LV), known to enhance the efficacy of 5-FU by forming a ternary complex with 5-FU metabolite and thymidylate synthase (TS) which prolongs the inhibition of TS and blocks DNA synthesis. It was proved UFT, a third-generation oral 5-FU, had favorable activity and tolerability when combined with LV in AGC patients [3]. A phase II study of S-1 plus LV (S-1/LV) has demonstrated promising efficacy and acceptable safety in patients with metastatic colorectal cancer (mCRC) [4]. A randomized phase II study reported promising efficacy of S-1/LV and S-1/LV plus oxaliplatin (SOL) for AGC, compared with S-1 plus cisplatin [5]. However, there has been no one-arm phase I/II study for S-1/LV in gastric cancer patients. Interindividual variation in pharmacogenetics of the S-1 metabolic pathway can affect the extent of S-1 metabolism and impact the efficacy and toxicity of S-1-based chemotherapy. Published studies all used “candidate” pharmacogenetic factors to predict the outcomes of AGC patients with S-1 or S-1-based chemotherapy, none of which used S-1 pharmacogenetic pathway approach, which means none integrated CYP2A6 polymorphism, 5-FU metabolic enzymes, and pharmacokinetics at the same time [6–8]. However, those factors do not act in isolation. What is more, the results and methods varied across studies. Finally, predictive values of these candidates might or might not be overcome with drugs combined with S-1.

Preclinical studies have highlighted the importance of TS, the cellular target of 5-FU–folinic acid mechanism of action [9]. Low TS was reported as a predictor of high response and long survival for AGC patients with high-dose 5-FU/LV [10]. In colorectal cancer, both intratumoral TS and DPD gene expressions had been reported to be predictive for the effectiveness of 5-FU/LV or UFT/LV [11, 12]. However, there was no research on the prediction of OPRT, TS, TP, or DPD for efficacy or toxicity of S-1/LV or UFT/LV in gastric cancer. What is more, no available evidence existed on the predictive potential of CYP2A6 polymorphism for 5-FU/LV, UFT/LV, or S-1/LV treatment in gastric cancer. Interestingly, the use of more than single gene expression, such as the combination of low TS with high OPRT, or high OPRT/TS had been reported to be even more predictive of responders to S-1 or S-1 plus cisplatin in gastric cancer patients [13–15].

This current phase II study of S-1/LV is deemed necessary to explore its efficacy and safety as first-line chemotherapy for AGC patients. Considering the frequent grade 3 toxicities, dose reduction, and period prolongation in that previous trial of mCRC [4], the 4-week S-1/LV was modified as 2 week in this study. As the first one, this study used S-1 pharmacogenetic pathway approach and integrated CYP2A6 polymorphism, DPD, TS, OPRT, thymidine phosphorylase (TP), and 5-FU pharmacokinetics to identify the subset of patients benefiting more, suffering less from S-1/LV.

## Patients and methods

As a one-arm, single-center, open phase II clinical trial, it is approved by the independent Institute Research Ethics Committee at the Sun Yat-sen University Cancer Center and conducted in accordance with the Declaration of Helsinki.

### Patient selection

The eligibility criteria included (1) histologically confirmed metastatic or recurrent gastric cancer; (2) at least one measurable lesion; (3) an age of ≥18; (4) adequate oral intake; (5) no previous antitumor therapy within 5 years (adjuvant chemotherapy without S-1 was allowed if finished ≥6 months before enrollment); (6) an Eastern Cooperative Oncology Group performance status <2; and (7) adequate bone marrow, hepatic, and renal function.

The exclusion criteria included (1) known hypersensitivity to any of the study drugs, or usage of drugs interacting with S-1; (2) serious concomitant conditions; (3) extensive bone, brain, or meningeal metastasis; (4) another synchronous cancer; (5) surgery within 3 months; (6) participating in other clinical studies; (7) pregnant women; (8) subjects with reproductive potential who were unwilling to use an effective method of contraception.

### Treatment schedule

S-1 (20-mg capsules) and LV (25-mg tablets) were provided by DaPeng Co., Ltd, Japan. All patients were orally treated with S-1 in doses of 40 mg (body surface area (BSA) < 1.25 m^2^), 50 mg (1.25 ≤ BSA < 1.50 m^2^) and 60 mg (BSA ≥ 1.50 m^2^) b.i.d. in combination with LV given simultaneously at a fixed dose of 25 mg b.i.d. on days 1–7, followed by a 7-day rest.

### Response and adverse event assessment

Clinical and laboratory examinations were carried out within 7 days before enrollment and each cycle of chemotherapy afterward. Tumor measurement was conducted on the basis of computed tomographic scans, within 15 days before enrollment and every 3 cycles afterward, according to Response Evaluation Criteria in Solid Tumors guidelines (version 1.1). Patients were considered response-assessable if they had overt clinical or radiological evidence of early PD within the first three cycles. All treatment-related adverse events (AEs) were categorized according to the National Cancer Institute’s Common Terminology Criteria for Adverse Events version 4.0 (NCI-CTCAE v4.0).

### Blood specimens

Peripheral blood was prospectively, anonymously sampled for each patient on the first day of the first cycle at 0 h (pre-dose) and 0.5, 1, 2, 4, 8, 24 h post-S-1/LV morning dosing. The 2 ml plasma at 0 h was separated for measuring baseline protein expression level of DPD, OPRT, TS, and TP. The 4-ml blood cells at 0 h were separated for CYP2A6 gene polymorphism. The 2 ml plasma at 0, 0.5, 1, 2, 4, 8, 24 h was separated for plasma concentrations of 5-FU.

### DPD, OPRT, TS, TP, CYP2A6 gene polymorphism, and 5-FU pharmacokinetics

The plasmic protein expression level of DPD, OPRT, TS, and TP was determined by an enzyme-linked immunosorbent assay (ELISA), as described by Cui et al. [7].

Polymerase chain reaction (PCR)–restriction fragment length polymorphism were used to determine common variant alleles that affect CYP2A6 activity or expression in Asian population (CYP2A6*1A, *1D, *9), *13, and the wild-type allele (CYP2A6*1), as previously described [16].

Plasma concentrations of 5-FU at 0, 0.5, 1, 2, 4, 8, 24 h were measured using negative ion chemical ionization gas chromatography mass spectrometry. Pharmacokinetic parameters including area under the curve (AUC_0–24h_), maximum concentration (C_max_), time taken to reach maximum concentration (T_max_), half-time (T_1/2_), area under the first moment curve (AUMC_0–24h_), mean resistance time (MRT_0–24h_), and plasma clearance (CL) were derived with non-compartmental methods using WinNonlin version 3.1 [17].

### Statistical analysis

The primary endpoint of this study was overall response rate (ORR), while the secondary endpoints were progression-free survival (PFS), time to treatment failure (TTF), overall survival (OS), disease control rate (DCR), and adverse events (AEs).

The sample size for the study was calculated from an expected response rate of 40–45% and threshold response rate of 20% with α = 0.05 and β = 0.2. Therefore, 31–45 patients were required in this study.

The Kaplan–Meier method with two-sided log-rank test was used to estimate the distribution of time to events. PFS was determined from the date of treatment to progression (PD) or death from cancer. TTF was determined from the date of treatment to PD, death, refusal, or interruption due to AEs. OS was calculated from the date of treatment to death from any cause or the last date of follow-up. Receiver operating characteristic (ROC) curve was used for cutoff values of DPD, OPRT, TP, TS, and 5-FU pharmacokinetics in the predictive analyses of response or grade 3–4 AEs. X-Tile software was used for cutoff values of them in the predictive analyses of survival. Logistic regression was used for predictive analyses of response or grade 3–4 AEs, and Cox proportional hazards model was used for predictive analyses of survival. Statistical analyses were performed using the SPSS 19.0.

## Results

### Patient characteristics

Between July 2011 and July 2012, a total of 39 eligible patients were enrolled from the Sun Yat-sen University Cancer Center. Clinical cutoff date was March 20, 2014. The median follow-up was 23.13 months. The baseline patient characteristics are summarized in Table 1. The median number of treatment cycles was 6 (range 1–20), with a total of 252. The median treatment period was 3.03 months (range 0.47–12.00). The median relative dose intensity was 91% for S-1 and 100% for LV.

**Table 1:** Baseline patient characteristics

Characteristics	S-1 plus LV (*N* = 39)	
	No.	%
Gender (male/female)	28/11	71.8/28.2
Age (years, median, range)	55 (21–83)	
Body surface area (m^2^, median, range)	1.51 (1.33–1.94)	
ECOG performance status = 1	39	100
Primary tumor location		
Proximal	11	28.2
Body	7	17.9
Antrum	10	25.6
Multiple/diffuse	11	28.2
Histology		
Well differentiated	3	7.7
Moderately differentiated	7	17.9
Poorly differentiated	24	61.5
Mucinous	4	10.3
Signet-ring cell	1	2.6
Lauren classification		
Diffuse type	4	11.1
Intestinal type	13	33.3
Mixed type	22	55.6
Her-2 gene type		
Positive	6	15.4
Negative	33	84.6
Site of metastases		
Liver	14	35.9
Lung	3	7.7
Lymph nodes	29	74.4
Peritoneum	14	35.9
Bone	4	10.3
No. of metastatic/recurrent sites		
1	18	46.2
2	16	41.0
3	5	12.8
Prior surgery		
Curative gastrectomy	8	20.5
Palliative gastrectomy/metastectomy	5	12.9
Exploration/bypass	4	10.3
No	22	56.4
Prior adjuvant chemotherapy		
Yes	6	15.4
No	33	84.6

### Overall response rate

All 39 patients were evaluable. No patient had a complete response, 16 had partial response (PR), 14 had stable disease, and 9 had progressive disease. The ORR was 41.0% (95% confidence interval (CI) 24.9–57.2%), and the DCR was 76.9% (95% CI 63.1–90.8%). The median time to response was 1.70 (range 1.40–3.00) months (m).

### Survival

**Fig. 1:**
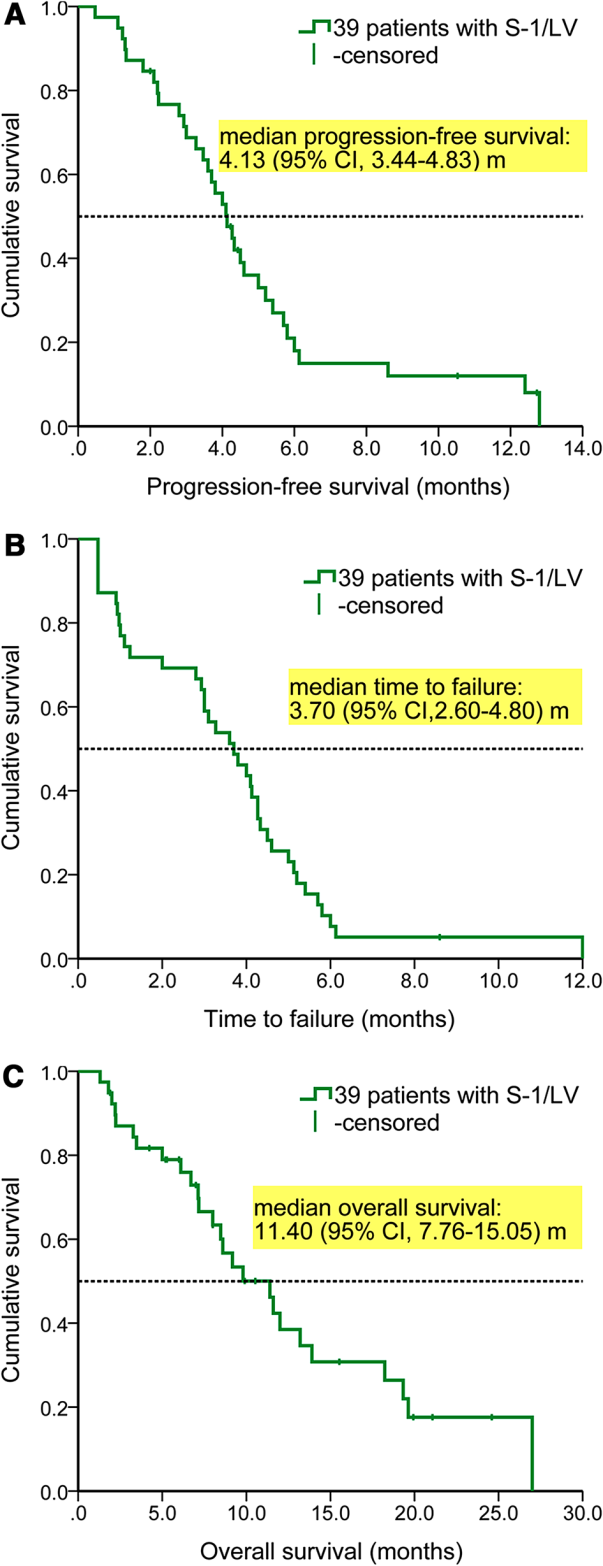
Kaplan–Meier curves for the entire population. **a** Progression-free survival, **b** time to failure, and **c** overall survival

The median PFS was 4.13 (95% CI 3.44–4.83) m. The median TTF was 3.70 (95% CI 2.60–4.80) m. The median OS was 11.40 (95% CI 7.76–15.05) m (Fig. 1).

### Adverse events, feasibility, and compliance of treatment

Major AEs included myelosuppression (74.4%), gastrointestinal reactions (89.7%), and pigmentation (53.8%); however, they were generally mild and no treatment-related deaths occurred. Anemia (71.8%) was common, and thrombocytopenia was rare (0%). Anorexia (64.1%) was common, and diarrhea was not. Grade 3 AEs occurred in 13 patients (33.3%), and grade 4 AEs occurred in 1 of them (2.6%). Each type of the grade 3–4 AEs occurred in only 1–3 patients, with gastrointestinal reactions in 15.4% and myelosuppression in 10.3% (Table 2).

**Table 2:** Adverse events

Adverse events	S-1 plus LV (*N* = 39)				
	Grade 1 (%)	Grade 2 (%)	Grade 3 (%)	Grade 4 (%)	All grade (%)
Leukopenia	6 (15.4)	7 (17.9)	1 (2.6)	0	14 (35.9)
Neutropenia	4 (10.3)	7 (17.9)	1 (2.6)	1 (2.6)	12 (33.3)
Anemia	17 (43.6)	10 (25.6)	1 (2.6)	0	28 (71.8)
Thrombocytopenia	0	0	0	0	0
Asthenia	9 (23.1)	0	0	0	9 (23.1)
Anorexia	19 (48.7)	5 (12.8)	1 (2.6)	0	25 (64.1)
Nausea	11 (28.2)	1 (2.6)	0	0	12 (30.8)
Vomiting	3 (7.7)	2 (5.1)	1 (2.6)	0	6 (15.4)
Diarrhea	9 (23.1)	1 (2.6)	1 (2.6)	0	11 (28.2)
Abdominal pain	5 (12.8)	4 (10.3)	2 (5.1)	0	11 (28.2)
Skin rash	5 (12.8)	0	0	0	5 (12.8)
Hand–foot syndrome	3 (7.7)	1 (2.6)	0	0	4 (10.3)
Pigmentation	16 (41.0)	5 (12.8)	0	0	21 (53.8)
Stomatitis	6 (15.4)	3 (7.7)	1 (2.6)	0	10 (25.6)
Blurred vision	4 (10.3)	0	0	0	4 (10.3)
Lacrimation increased	4 (10.3)	0	0	0	4 (10.3)
Tinnitus	1 (2.6)	0	0	0	1 (2.6)
ALT elevation	4 (10.3)	2 (5.1)	3 (7.7)	0	9 (23.1)
AST elevation	4 (10.3)	1 (2.6)	2 (5.1)	0	7 (17.9)
Hypoalbuminemia	12 (30.8)	2 (5.1)	0	0	14 (35.9)
Proteinuria	1 (2.6)	0	0	0	1 (2.6)

The main reasons for S-1 dose decrease in the 3 patients (7.7%) were grade 3 diarrhea, anorexia, and stomatitis, respectively. The main reasons for dose interruption in the 3 patients (7.7%) were grade 3 vomiting, abdominal pain, and liver enzyme elevation, respectively. The median number of chemotherapy cycles before S-1 dose decrease and interruption was 4 (range 2–6) and 5 (range 2–10), respectively. Course was prolonged by 7 days until the grade 3 liver enzyme elevation decreased to grade 1 in 1 patient. Five patients (12.8%) discontinued treatment before progression not due to AEs, with a median number of treatment cycles as 3 (range 1–6) (supplementary Table S1). Second-line treatment was given to 21 (53.9%) of the 39 patients, among whom 5.1% received palliative surgery while 48.8% received oxaliplatin-based, irinotecan-based, or taxane-based chemotherapy (supplementary Table S2).

### Clinical prediction of efficacy and toxicity

For the entire population, baseline plasmic protein expression of DPD, OPRT, TP, TS and their ratios OPRT/DPD, OPRT/TP, OPRT/TS, OPRT/TP + TS, OPRT/DPD + TP, OPRT/DPD + TS, and OPRT/DPD + TS + TP are summarized in supplementary Table S3. The genotypes and allele frequencies of CYP2A6 are shown in supplementary Table S4. Mean plasma concentration–time curve of 5-FU for the entire population is shown in supplementary Figure S1. The AUC_0–24h_, C_max_, T_max_, T_1/2_, AUMC_0–24h_, MRT_0–24h_, CL were determined for each patient and for the entire population.

#### Prediction of response

**Fig. 2:**
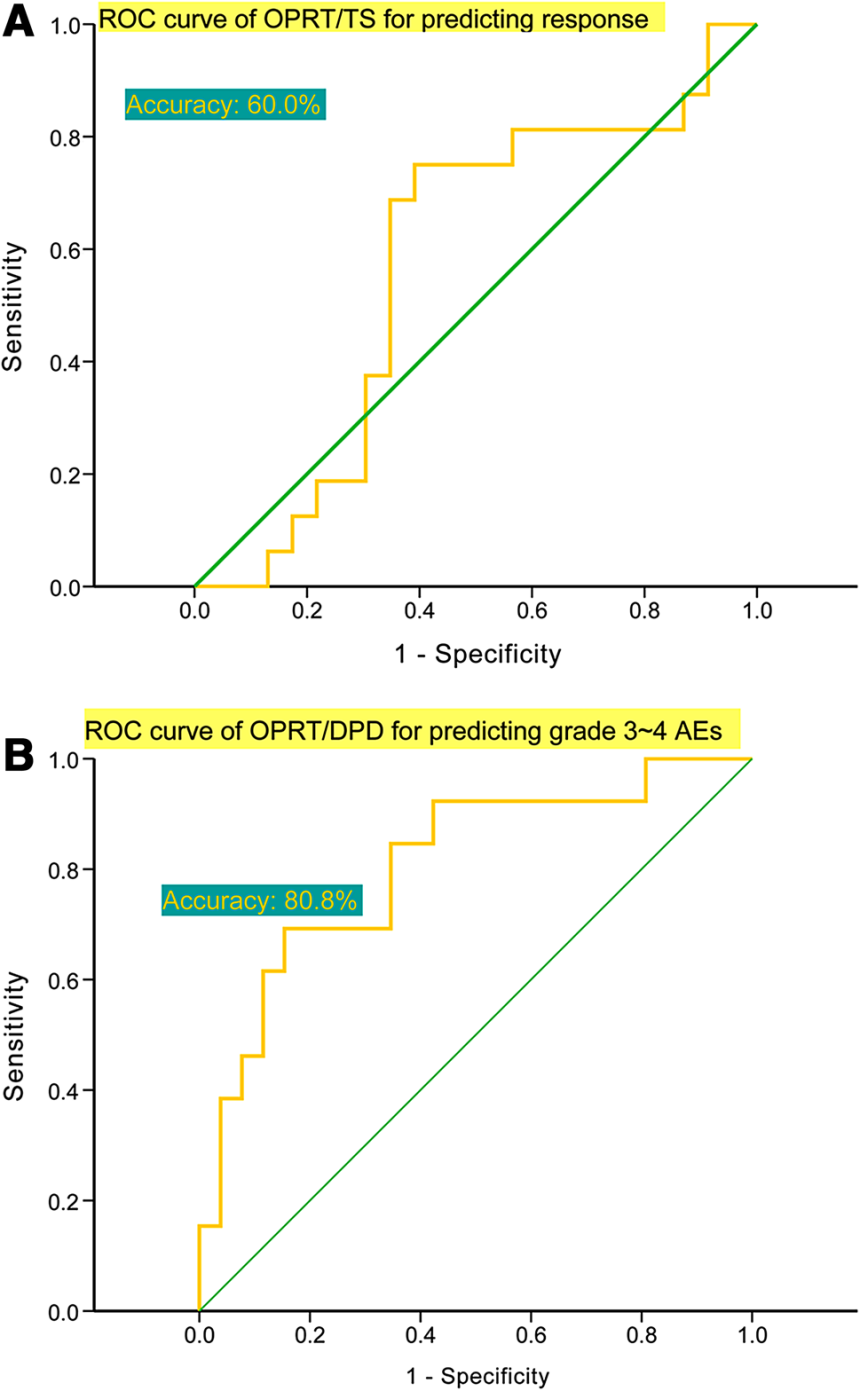
**a** ROC curve of OPRT/TS for predicting response and **b** the ROC curve of OPRT/DPD for predicting grade 3–4 AEs. *ROC* receiver operating characteristic, *OPRT* orotate phosphoribosyltransferase, *TS* thymidylate synthase, *DPD* dihydropyrimidine dehydrogenase, *AEs* adverse events

There were 16 responders and 23 non-responders. By multivariate logistic regression analysis, high OPRT/TS (>1.246 vs. ≤1.246, odds ratio (OR) 16.962, 95% CI 1.781–161.581, *P* = 0.014) and peritoneal metastasis (vs. liver metastasis, OR 25.604 (1.852–353.979), *P* = 0.016) were independently predictive of responding. OPRT/TS differed between responders and non-responders (median ± SD 1.442 ± 0.091 vs. 1.158 ± 0.133, *P* = 0.037) and response rates differed between patients with high OPRT/TS and low OPRT/TS (>1.246 vs. ≤1.246, 57.1 vs. 22.2%, *P* = 0.040). Figure 2a shows the ROC curve of OPRT/TS for predicting response.

#### Prediction of grade 3–4 adverse events

Thirteen patients experienced grade 3–4 AEs and 26 patients did not. By univariate logistic regression analysis, high OPRT (accuracy 79.0%), high OPRT/DPD (80.8%), high OPRT/TP (77.8%), high OPRT/TS (71.0%), high OPRT/DPD + TP + TS (78.7%), high OPRT/DPD + TS (77.8%), high OPRT/TP + TS (77.2%), and high OPRT/DPD + TP (80.1%) were all associated with grade 3–4 AEs. OPRT/DPD exhibited the highest accuracy (80.8%). By multivariate logistic regression analysis, high OPRT/DPD [>0.754 vs. ≤0.754, OR 15.566 (1.490–162.605), *P* = 0.022] was independently predictive of grade 3–4 AEs. The rates of grade 3–4 AEs differed between patients with high OPRT/DPD and low OPRT/DPD (>0.754 vs. ≤0.754, 55.0 vs. 10.5%, *P* = 0.006). Figure 2b shows the ROC curve of OPRT/DPD for predicting grade 3–4 AEs.

#### Prediction of progression-free survival and time to failure

Multivariate analysis with a Cox proportional hazards model demonstrated that high AUC_0–24h_ of 5-FU and metastatic/recurrent sites ≤2 (vs. >3) were significant predictors of prolonged PFS (supplementary Table S5). Similarly, multivariate analysis demonstrated high AUC_0–24h_ of 5-FU was borderline significant predictor of prolonged TTF (supplementary Table S6).

**Fig. 3:**
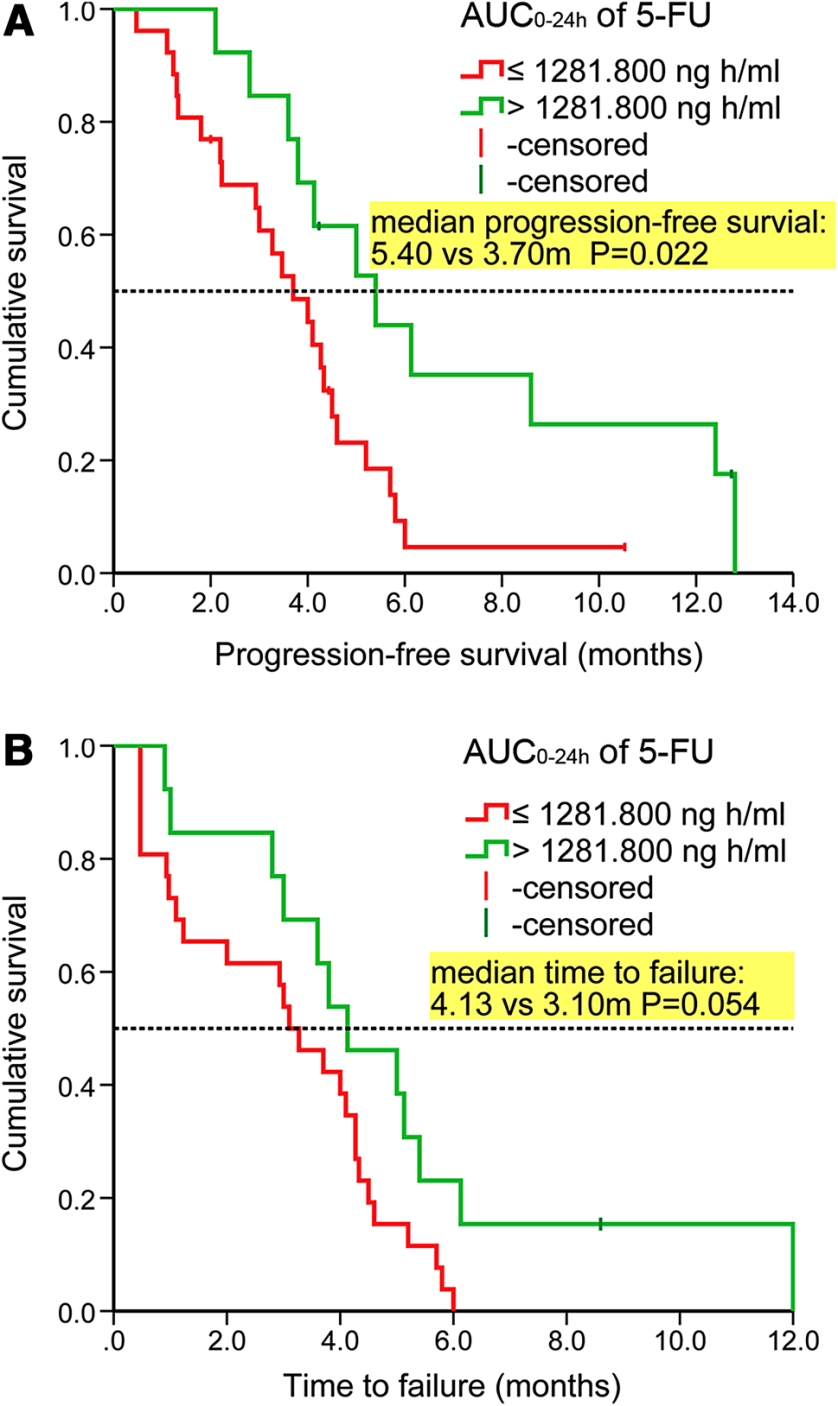
**a** Kaplan–Meier curve of progression-free survival according to AUC_0–24h_ of 5-FU and **b** the Kaplan–Meier curve of time to failure according to AUC_0–24h_ of 5-FU. *AUC* areas under the curve

The median PFS differed significantly between patients with high and low AUC_0–24h_ of 5-FU (5.40 vs. 3.70 m, *P* = 0.022, Fig. 3a), and the median TTF differed borderline between patients with high and low AUC_0–24h_ of 5-FU (4.13 vs. 3.10 m, *P* = 0.054, Fig. 3b).

#### Prediction of overall survival

**Fig. 4:**
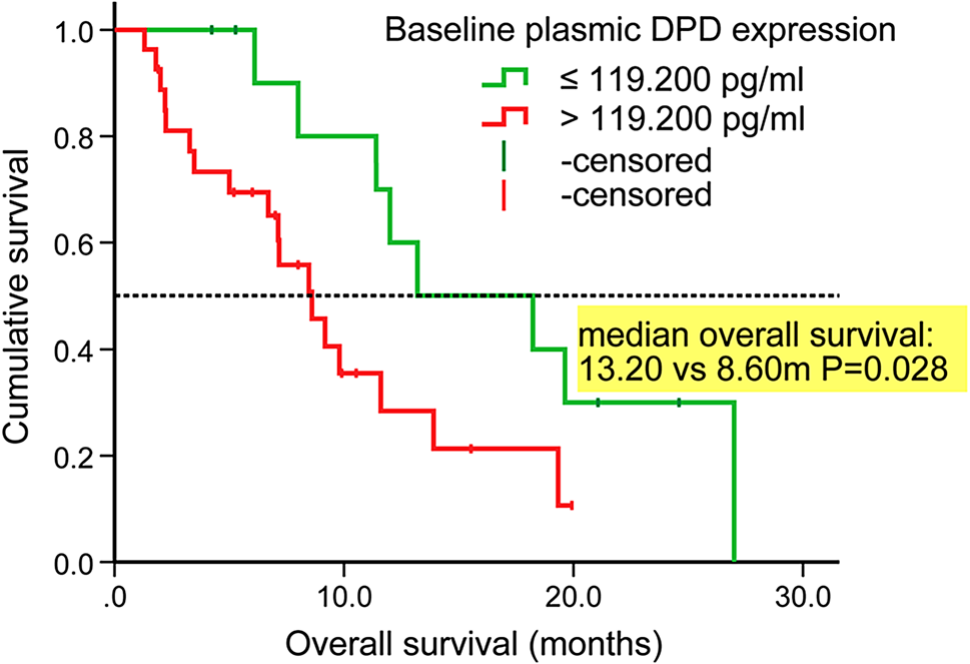
Kaplan–Meier curve of overall survival according to baseline plasmic DPD expression. *DPD* dihydropyrimidine dehydrogenase

Lower baseline plasmic DPD [>119.200 vs. ≤119.200, harzard ratio (HR) 2.931 (1.155–7.433), *P* = 0.024] was significantly independent predictor of prolonged OS (supplementary Table S7; Fig. 4).

To integrate the above predictors, we classified 39 patients into 4 patients with high OPRT/TS, high AUC_0–24h_ of 5-FU, low DPD (ORR 50.0%, median PFS 7.00 m, median OS 13.90 m), and other 35 patients (ORR 40.0%, median PFS 4.10 m, median OS 9.17 m).

## Discussion

To our knowledge, the current study is the first one-arm and the second phase II trial to evaluate efficacy and toxicity of S-1/LV chemotherapy in AGC patients, which is the first to predict outcomes using S-1 pharmacogenetic pathway approach, on the basis that LV is but a cofactor entering the 5-FU metabolism. The enrollment was between July 2011 and July 2012, which was similar to between October 2011 and December 2012 of that randomized phase II study of S-1/LV versus SOL versus SP for AGC [5].

We reported S-1/LV regimen yielded promising ORR, PFS, TTF, and OS, without combination with platinum, taxane, irinotecan, or trastuzumab as first-line treatment. In previous phase II studies of S-1 monotherapy in AGC and previous S-1 monotherapy arms of phase III JCOG9912 study, SPIRITS study, SC-101 study, GC0301/TOP-002 study, and START study, conventional dose for 4 weeks followed by 2-week rest was usually given [18–22]. The 2-week S-1/LV here was generally more effective, with a less dose intensity than S-1 monotherapy in these studies. LV can enhance the efficacy of 5-FU by maintaining the plasma 5-FU concentration [23]. Similarly with the previous trial of S-1/LV in mCRC [4], S-1/LV regimen here also demonstrated better efficacy and safety compared with previously reported UFT/LV in AGC [3, 24]. S-1/LV also showed more potential than 5-FU/LV plus oxaliplatin that we reported in terms of efficacy and safety [25].

To reduce the duration of effective drug concentration and keeping an appropriate rest period could be effective method of improving safety. So we modified the schedule as 1-week S-1/LV, followed by 1-week rest period, the same as S-1/LV group in that randomized phase II study. The efficacy S-1/LV here was quite comparable with S-1/LV group in that randomized phase II study, in terms of ORR (41.0 vs. 43%), PFS (4.13 vs. 4.2 m), and TTF (3.70 vs. 4.1 m) [5]. Fewer patients in our study received the second-line treatment (53.9 vs. 77%), which helped explain the relatively not so promising OS. Another reason was that there were 5 patients discontinuing treatment before progression not due to adverse events. Meanwhile, S-1/LV in this study saw very satisfactory safety. The frequencies of each type of AEs were generally lower than those in the phase II trial of mCRC and comparable with those in the randomized phase II trial of AGC [4, 5]. Encouragingly, grade 4 AEs occurred in 1 patient (2.6% neutropenia), similarly for the randomized study (2% grade 4 neutropenia and 2% leucopenia). Each type of the grade 3 AEs occurred in only 2.6–7.7% patients, similar to 2–13% for the randomized study [5]. In our study, significantly fewer patients experienced dose reduction, or delayed courses [4], compared to patients with S-1 monotherapy in previous phase III trials [19, 26]. In our study, gastrointestinal reactions were more common than myelosuppression both in total (89.7 vs. 74.4%) and in grade 3–4 (15.4 vs. 10.3%), and dose reductions or interruptions were chiefly due to diarrhea, stomatitis, anorexia, and vomiting. We also observed dose-limiting toxicity was shifted from hematological to gastrointestinal when S-1 was administered with LV [4]. Probably the capacity of oral intake and gastrointestinal tolerability would be the important indications for this regimen.

Japan Gastric Cancer Association guideline states that S-1 alone could be considered for patient who is not suitable for S-1 plus cisplatin therapy [27]. Previous studies showed S-1 monotherapy could be a reasonable option in the treatment of elderly patients. In our study, the ORR, PFS, OS were sound for patients of age >70 (47.1%, 6.00, 11.40 m) without difference compared with those of age ≤70 (36.4%, 4.00, 11.20 m) and seemed better than previously reported S-1 monotherapy (ORR 14.3–26.3%) and UFT/LV (ORR 22%) for elderly AGC patients [28–30]. The oral convenience makes the S-1/LV regimen extremely useful clinically, especially for elderly patients.

Genotyping the peripheral blood for CYP2A6 polymorphism, quantifying plasmic protein expression of DPD, TS, TP, and OPRT with ELISA, is more optimal, convenient, quicker than evaluating genes, mRNAs, or proteins in tumor tissue, especially when 5-FU plasmic pharmacokinetics were to be integrated at the same time. Thus, there may be more clinical accessibility and prospects. What is more, the cutoff levels were determined by standard statistic analysis, not the simple median or mean. The plasmic expression of DPD in this study was consistent with previously reported with ELISA; however, no report for plasmic OPRT, TP, or TS was available [7]. The frequencies of CYP2A6 alleles in this population were compatible with other Asian population [31–33]. We especially examined the CYP2A6*13, because its function in Asian patients was unclear. The 0% of CYP2A6*13 again proved it is rare in Asia.

In clinical setting, numerous studies have reported a low TS, low TP, or a high OPRT expression contributed to a high sensitivity to UFT, S-1, or S-1-based treatment in gastric cancer patients, with or without influencing the PFS or OS [34–38]. There were also evidences that low TS was a predictor of high response for AGC patients with 5-FU/LV, or 5-FU/LV plus cisplatin/oxaliplatin [10, 39, 40]. However, most studies failed to find the DPD expression related to either response, PFS, or OS and less studies did demonstrate high DPD mRNA was predictor of poor OS, not ORR or PFS in AGC [35, 41, 42]. These clinical findings reflect theoretical roles of TS and OPRT. We found high OPRT/TS alone significantly predicted responding. The resultant high OPRT/TS here revealed preferential use of the OPRT pathway versus TS pathway during 5-FU metabolism. In humans, the preferential use of the OPRT pathway was revealed to correlate with a higher sensitivity to 5-FU [43]. Ichikawa et al. and Tanemura et al. [13, 14] both reported the combination of high OPRT and low TS was more predictive of responders to S-1 or S-1 based chemotherapy in gastric cancer patients than either alone, while the low TP was not. Apart from the above PCR or ELISA methods, the quantitative double-fluorescence immunohistochemistry method, reported by Hashiguchi K, was used to access the protein expressions and their ratios quantitatively and found a significant correlation between OPRT/TS, OPRT/DPD, or OPRT/(TS + DPD) and response to S-1 in the AGC patients, among which OPRT/TS showed the strongest correlation with the clinical response [15]. These three studies generally agree with our finding.

In this current study, low baseline plasmic DPD was not related to response; however, it was related to long OS, compatible with previous study that high intratumoral DPD mRNA was predictor of poor OS, not ORR or PFS [41]. Previous studies reported clinical response to S-1 in gastric cancer was not influenced by intratumoral DPD expression [42]. It can be explained that S-1 has antitumor activity even in tumor with high expression of DPD because of the inhibition of DPD by CDHP [44]. Even though it had no prediction of response, low DPD did relate to long OS. Firstly, S-1 enabled high 5-FU concentrations to be maintained in blood for long periods of time by inhibiting of DPD and 5-FU maintenance was a reason of long survival. Secondly, low levels of intratumoral DPD have been generally shown to predict long survival in gastric cancer patients treated with 5-FU-based chemotherapy [45]. Many patients here received second-line chemotherapy comprising of 5-FU with platinum or other drugs.

We found plasmic OPRT (*P* = 0.024), TS (*P* = 0.044) expression significantly inversely, while DPD (*P* = 0.073), TP (*P* = 0.080) borderline inversely correlated with AUC_0–24h_ of 5-FU. The number of CYP2A6 gene variants (*P* = 0.889) did not correlate with AUC_0–24h_. This was consistent with that CYP2A6 gene correlated with tegafur pharmacokinetics, but not with 5-FU pharmacokinetics [16]. AUC_0–24h_ of 5-FU, not OPRT/TS, was predictive of PFS revealed that other factors may also influence PFS by AUC_0–24h_ of 5-FU. We did not demonstrate the number of CYP2A6 gene variants correlated with efficacy. Although some studies demonstrated patients having fewer CYP2A6 variants had better PFS in AGC patients with S-1 plus cisplatin, or S-1 plus docetaxel [6, 46], divergences on the relation between CYP2A6 genetic polymorphisms and response existed for both gastric and colorectal cancer patients with S-1 or S-1-based chemotherapy [47, 48]. In our study, second-line treatment excluded S-1 and tegafur. CYP2A6 converts enzymatically tegafur, the effector molecule of S-1, to 5-FU, and this role produces no meaning on second-line treatment.

That high OPRT predicted grade 3–4 AEs as well as affected response can be theoretically understood, and in animal models, oteracil in S-1 was found to inhibit the OPRT by 70% in the small intestine; however, the inhibition was limited to 0–20% in tumor regions without affecting the antitumor activity of 5-FU. Besides, high OPRT/DPD, OPRT/TP, OPRT/TS, OPRT/DPD + TP + TS, OPRT/DPD + TS, OPRT/TP + TS, and OPRT/DPD + TP were all associated with grade 3–4 AEs, among which, high OPRT/DPD exhibited the highest accuracy. Cui et al. [7] reported lower baseline plasmic DPD correlated with higher grade of toxicities in AGC patients with S-1 plus docetaxel by ELISA. Further studies were warranted to decide whether OPRT or OPRT/DPD better predicts grade 3–4 AEs. The literature shows severe diarrhea was dose-limiting toxicity for S-1 in Caucasians and severe neutropenia in East Asians perhaps due to CYP2A6 gene polymorphism [17], while this study did not see its relation to severe diarrhea, neutropenia, or total grade 3–4 AEs and no relation of CYP2A6 gene polymorphism to AUC_0–24h_ or C_max_ of 5-FU here helped explain.

## Conclusion

The 2-week S-1/LV regimen demonstrated promising efficacy and satisfactory safety as first-line chemotherapy for AGC. To balance both the efficacy and toxicity, S-1 pharmacogenetic pathway may help find an optimal subset of patients with high OPRT/TS, high AUC_0–24h_ of 5-FU, low DPD that may benefit more from S-1/LV, which awaits validation in another large and well-defined population.

## Electronic supplementary material

Below is the link to the electronic supplementary material.

Fig. S1 Mean plasma concentration–time curve of 5-FU for the entire population. 5-FU: fluorouracil, AUC_0-24h_: areas under the curve, C_max_: maximum concentration, T_max_: time taken to reach maximum concentration, T_1/2_: half-time, AUMC_0-24h_: area under the first moment curve, MRT_0-24h_: mean resistance time, CL: plasma clearance (TIFF 163 kb)

Supplementary material 2 (DOCX 19 kb)
