# Supplementary figures and images for: Phase II trial of S-1 plus leucovorin in patients with advanced gastric cancer and clinical prediction by S-1 pharmacogenetic pathway
Source: Cancer Chemother Pharmacol. 2016 Dec 2;79(1):69–79. doi: 10.1007/s00280-016-3209-1 (PMC5225176; doi:10.1007/s00280-016-3209-1)

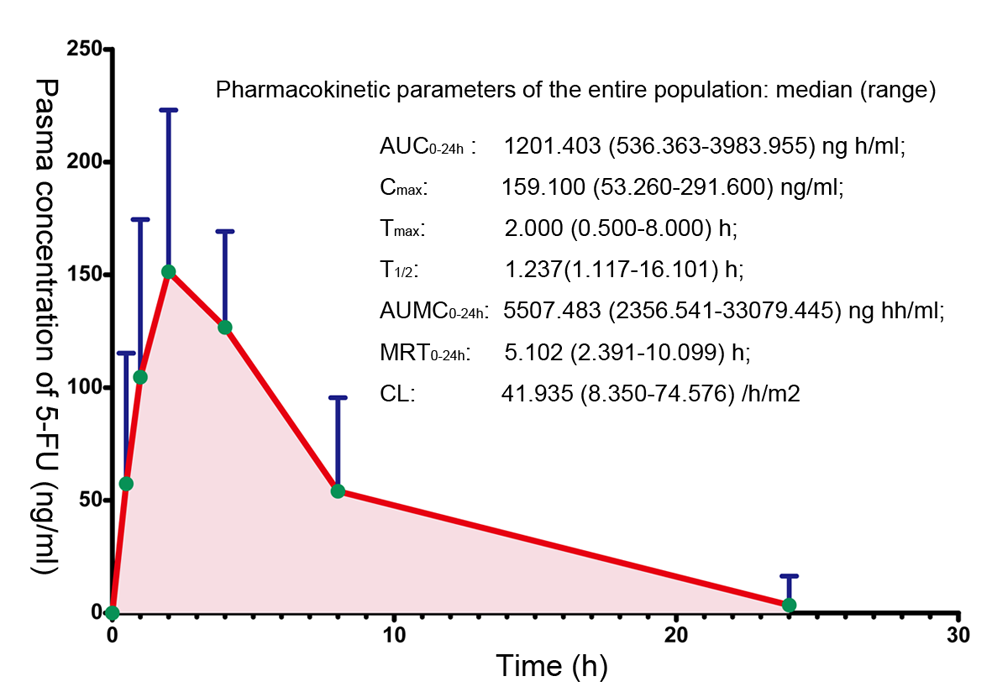

Supplement: Supplementary file 1 — Mean plasma concentration–time curve of 5-FU for the entire population. 5-FU: fluorouracil, AUC0-24h: areas under the curve, Cmax: maximum concentration, Tmax: time taken to reach maximum concentration, T1/2: half-time, AUMC0-24h: area under the first moment curve, MRT0-24h: mean resistance time, CL: plasma clearance (TIFF 163 kb) [file 280_2016_3209_MOESM1_ESM.tif]
