# Supplementary material for: Phase II trial of S-1 plus leucovorin in patients with advanced gastric cancer and clinical prediction by S-1 pharmacogenetic pathway
Source: Cancer Chemother Pharmacol. 2016 Dec 2;79(1):69–79. doi: 10.1007/s00280-016-3209-1 (PMC5225176; doi:10.1007/s00280-016-3209-1)
Supplement: Supplementary file 2 — Supplementary material 2 (DOCX 19 kb) [file 280_2016_3209_MOESM2_ESM.docx]

**Table S1**  Compliance and feasibility

|  | S-1 plus LV (*N* = 39) | |
| --- | --- | --- |
|  | No. | % |
| Patients following planned schedule and dose | 27 | 69.2 |
| Patients with interruption due to adverse events | 3 | 7.7 |
| Patients with dose-reduction | 3 | 7.7 |
| Patients with delayed courses | 1 | 2.5 |
| Patients discontinued not due to adverse events | 5 | 12.8 |

**Table S2**  Further treatment after study chemotherapy

|  | S-1 plus LV (*N* = 39) | |
| --- | --- | --- |
|  | No. | % |
| Oxaliplatin based | 4 | 10.3 |
| Irinotecan based | 1 | 2.6 |
| Docetaxel/ paclitaxel based | 8 | 20.5 |
| Abraxane based | 6 | 15.4 |
| Palliative surgery | 2 | 5.1 |
| None | 18 | 46.1 |

**Table S3**  Baseline plasmic protein expression of DPD, OPRT, TP, TS and their ratios

|  | Median | Range |
| --- | --- | --- |
| DPD (pg/ml) | 134.351 | 100.743-354.964 |
| OPRT (pg/ml) | 116.741 | 72.290-276.465 |
| TP (ng/ml) | 134.592 | 124.179-158.315 |
| TS (pg/ml) | 89.820 | 64.580-182.360 |
| OPRT/DPD | 0.901 | 0.326-1.978 |
| OPRT/TP | 0.880 | 0.575-2.225 |
| OPRT/TS | 1.371 | 0.635-3.125 |
| OPRT/TP+TS | 0.506 | 0.314-1.300 |
| OPRT/DPD+TP | 0.433 | 0.242-1.047 |
| OPRT/DPD+TS | 0.550 | 0.216-1.211 |
| OPRT/DPD+TS+TP | 0.330 | 0.175-0.784 |

DPD: dihydropyrimidine dehydrogenase, OPRT: orotate phosphoribosyltransferase, TP: thymidine phosphorylase, TS: thymidylate synthase.

**Table S4**  Frequencies of CYP2A6 genotypes and alleles in the patients

|  | Frequency (n, %) | |
| --- | --- | --- |
| Allele |  |  |
| 1 | 21 | 26.9 |
| 1A | 13 | 16.7 |
| 1D | 27 | 34.6 |
| 9 | 17 | 21.8 |
| 13 | 0 | 0 |
| Genotype |  |  |
| CYP2A6*1/CYP2A6*1 | 1 | 2.6 |
| CYP2A6*1/CYP2A6*1A | 5 | 12.9 |
| CYP2A6*1/CYP2A6*1D | 11 | 28.2 |
| CYP2A6*1/CYP2A6*9 | 3 | 7.7 |
| CYP2A6*1A/CYP2A6*1D | 5 | 12.9 |
| CYP2A6*1D/CYP2A6*9 | 11 | 28.2 |
| CYP2A6*1A/CYP2A6*9 | 3 | 7.7 |

**Table S5** Univariate and multivariate analysis of progression-free survival

| Variates | Univariate analysis | | | Multivariate analysis | | |
| --- | --- | --- | --- | --- | --- | --- |
|  | HR | 95% CI | *P* | HR | 95% CI | *P* |
| Metastatic/recurrent sites |  |  | 0.040 |  |  | 0.041 |
| ≤ 2 | 1 | reference |  | 1 | reference |  |
| 3 | 2.728 | 1.049-7.092 |  | 2.693 | 1.041-6.965 |  |
| AUC_0-24h_ of 5-FU |  |  | 0.026 |  |  | 0.027 |
| ≤ 1281.800 | 1 | reference |  | 1 | reference |  |
| > 1281.800 | 0.404 | 0.182-0.898 |  | 0.406 | 0.182-0.902 |  |

**Table S6** Univariate and multivariate analysis of time to failure

| Variates | Univariate analysis | | | Multivariate analysis | | |
| --- | --- | --- | --- | --- | --- | --- |
|  | HR | 95% CI | *P* | HR | 95% CI | *P* |
| Metastatic/recurrent sites |  |  | 0.013 |  |  | 0.477 |
| ≤ 2 | 1 | reference |  | 1 | reference |  |
| 3 | 2.487 | 1.210-5.019 |  | 1.391 | 0.559-3.460 |  |
| AUC_0-24h_ of 5-FU |  |  | 0.051 |  |  | 0.066 |
| ≤ 1281.800 | 1 | reference |  | 1 | reference |  |
| > 1281.800 | 0.492 | 0.234-1.033 |  | 0.498 | 0.237-1.048 |  |

**Table S7** Univariate and multivariate analysis of overall survival

| Variates | Univariate analysis | | | Multivariate analysis | | |
| --- | --- | --- | --- | --- | --- | --- |
|  | HR | 95% CI | *P* | HR | 95% CI | *P* |
| Baseline CEA (continuous) | 1 | 1.000-1.001 | 0.041 | 1 | 1.000-1.001 | 0.070 |
| Baseline plasmic DPD |  |  | 0.009 |  |  | 0.024 |
| ≤ 119.200 pg/ml | 1 | reference |  | 1 | reference |  |
| > 119.200 pg/ml | 1.014 | 1.003-1.025 |  | 2.931 | 1.155-7.433 |  |
| Baseline plasmic TP |  |  | 0.033 |  |  | 0.089 |
| ≤ 137.900 ng/ml | 1 | reference |  | 1 | reference |  |
| > 137.900 ng/ml | 0.365 | 0.145-0.920 |  | 0.449 | 0.179-1.129 |  |

DPD: dihydropyrimidine dehydrogenase, TP: thymidine phosphorylase
